# Differences between patients with chronic widespread pain and local chronic low back pain in primary care - a comparative cross-sectional analysis

**DOI:** 10.1186/1471-2474-14-351

**Published:** 2013-12-13

**Authors:** Annika Viniol, Nikita Jegan, Corinna Leonhardt, Markus Brugger, Konstantin Strauch, Jürgen Barth, Erika Baum, Annette Becker

**Affiliations:** 1 Department of General Practice/Family Medicine, University of Marburg, Karl-von-Frisch-Str. 4, 35043 Marburg, Germany; 2 Institute of Medical Informatics, Biometry and Epidemiology, Chair of Genetic Epidemiology, Ludwig-Maximilians-Universität, Ingolstädter Landstr 1, Munich 85764,Neuherberg, Germany; 3 Institute of Genetic Epidemiology, Helmholtz Zentrum München – German Research Center for Environmental Health, Ingolstädter Landstr 1, 85764 Neuherberg, Germany; 4 Institute for Social and Preventive Medicine (ISPM), University Bern, Niesenweg 6, 3012 Bern, Switzerland

**Keywords:** Chronic low back pain, Chronic widespread pain, Chronic localized pain

## Abstract

**Background:**

Chronic pain is a common reason for consultation in general practice. Current research distinguishes between chronic localized pain (CLP) and chronic widespread pain (CWP). The aim of this study was to identify differences between CWP and chronic low back pain (CLBP), a common type of CLP, in primary care settings.

**Methods:**

Fifty-eight German general practitioners (GPs) consecutively recruited all eligible patients who consulted for chronic low back pain during a 5-month period. All patients received a questionnaire on sociodemographic data, pain characteristics, comorbidities, psychosomatic symptoms, and previous therapies.

**Results:**

GPs recruited 647 eligible patients where of a quarter (n = 163, 25.2%) met the CWP criteria according to the American College of Rheumatology. CWP patients had significantly more comorbidities and psychosomatic symptoms, showed longer pain duration, and suffered predominantly from permanent pain instead of distinguishable pain attacks. CWP patients were more often females, are less working and reported a current pension application or a state-approved grade of disability more frequently. We found no other differences in demographic parameters such as age, nationality, marital status, number of persons in household, education, health insurance status, or in health care utilization data.

**Conclusions:**

This project is the largest study performed to date which analyzes differences between CLBP and CWP in primary care settings. Our results showed that CWP is a frequent and particularly severe pain syndrome.

**Trial registration:**

German Clinical Trial Register, DRKS00003123.

## Background

Chronic pain is a common reason for consultation in general practice. Current research distinguishes between chronic localized pain (CLP) and chronic widespread pain (CWP). CLP is defined as chronic pain restricted to one or few body regions (e.g. head, back, or knee). In contrast, CWP means chronic pain in several body regions. The American College of Rheumatology (ACR) defines CWP as pain in the left and right side of the body, as well as above and below the waist plus pain in the axial skeleton [1]. It is still unclear whether CWP is a complication of CLP or an independent pain syndrome [2].

Population based studies reported a CWP point-prevalence of 4.7%-15% depending on the country [3-8]. Nordeman et al. found a CWP prevalence of 28% in a sample of female chronic low back pain (CLBP) patients consulting primary care in Sweden [9]. The high CWP prevalence and the associated high social and economic burden for patients and health care systems [10] illustrate the need for further knowledge about CWP.

Several studies have investigated differences in physical and psychological factors between CWP patients and healthy adults: CWP patients are more frequently female, older, less educated [11], have a decreased health-related quality of life [7,11], are less physically active [12], and a receive disability pension more often [13] than patients without pain. They show a high prevalence of physical and psychological comorbidities [4,6,14-17] and an increased mortality risk [18].

In addition to differences in the pain distribution, it is also important to know if CWP patients are more negatively affected in other wellbeing areas when compared to CLP patients.

To date, few studies have examined differences between CWP and CLP. Two studies present population based data on neck-shoulder pain [19] and myofacial face pain [20]. They found a higher rate of pain intensity, pain duration, depression, and somatization symptoms among CWP patients. Among all CLP syndromes, CLBP is the most common [21]. Back pain is the second most frequent consultation reason in German primary care practices [22].

Only one study investigated differences of CLBP and CWP in primary care patients: Nordeman et al. [9] found an increased rate of activity limitation, impaired physical performance, work disability, severe pain, tender points, fatigue, depression, and severe clinical stress symptoms in CWP patients compared to CLBP patients. The authors also reported a lower health-related quality of life and a reduced private social support in CWP patients [9]. However, Nordeman et al. studied only women.

The aim of this study is to identify differences between CWP and chronic low back pain (CLBP), a common type of CLP, in primary care settings. This would enable us to suggest priorities for health care interventions in CWP.

## Methods

### Study design

The present study refers to the cross sectional baseline analysis of a 12-month cohort study which evaluates risk factors and protective factors of pain generalization in primary care CLBP patients. A detailed study protocol has been published elsewhere [23].

This project is part of the research consortium LOGIN “Localized and Generalized Musculoskeletal Pain: Psychobiological Mechanisms and Implications for Treatment” funded by the German Federal Ministry of Education and Research. The study was approved by the local ethics commission of Philipps University in Marburg, Germany (Ethik: 11.06.2010, AZ 88/10) and is in accordance with the Declaration of Helsinki.

### Study population

We invited all general practitioners (GPs) in the northern part of Germany’s state of Hessen to participate in our study. We asked participating GPs to consecutively recruit all eligible patients consulting for CLBP as a primary or secondary consulting reason (inclusion criteria), during a 5-month period. The symptom “chronic low back pain” was defined as pain in the back area under the costal arch, but over the bottom fold (with or without pain radiation), during most days in the last three months. Patients under 18 years, pregnant women, and patients with insufficient understanding of the German language or severe cognitive impairments (e.g., dementia) were excluded from the study.

### Data collection

Doctors asked patients for study participation directly after consultation. All participating patients received a pen and paper questionnaire. Patients who refrained from participation were asked to give reasons for their decline. During the recruitment period, trained clinical monitors conducted two random quality control checks of the GPs’ performance.

### Measurements

To explore distinctive features with regards to pain characteristics and sociodemographic data between CLBP and CWP patients, we evaluated the following physical and psychological parameters (for detailed information please see Viniol et al. [23]).

#### Pain characteristics and sociodemographic data

For definition of CLBP and CWP, we assessed pain localization with the body pain drawing model from Pfau et al. [24]. The CWP definition was derived from the ACR criteria from Wolfe et al. [1]. Pain anamnesis was assessed with the German Pain Questionnaire, the official pain questionnaire of the German Association for the Study of Pain [25]. We chose the modules referring to duration, characteristics, course of pain, sociodemographic data, health care utilization, and medication.

In addition, we used the 3-item social support subscale of the West Haven-Yale Multidimensional Pain Inventory (MPI) to explore the partner’s reaction in response to patient’s pain (internal consistency of the subscales: α = 0.63-0.90) [26,27]. We rated the severity of chronic pain with the German translation of von Korff’s Graded Chronic Pain questionnaire [28]. Severity is computed from “pain intensity” and “pain-related disability” (internal consistency of the subscales: α = 0.68-0.88). The lower range of the scale is determined by pain intensity; the higher range of the scale refers to pain intensity and pain-related disability [29].

#### Comorbidities

Using the Self-Administered Comorbidity Questionnaire, we asked the patients about 14 common medical conditions (high blood pressure, heart disease, asthma, chronic obstructive pulmonary disease, ulcer/stomach disease, diabetes, high blood lipid level, kidney disease, osteoarthritis/degenerative arthritis, rheumatoid arthritis, osteoporosis, cancer disease, depression, other psychiatric diseases) [8,30]. Patients were asked to specify if they suffer from any of these traits, whether they receive treatment for it, and whether it causes functional limitations.

#### Psychosomatic symptoms

The Symptom Checklist-90-R (SCL-90-R) assess typical physical symptoms which accompany functional dysfunctions, but could also arise in somatic disorders.

The SCL-90-R is a commonly used psychological status symptom inventory for psychopathology (internal consistency: α = 0.81) [31].

### General practitioners case report

Potential red flags, which are frequently used risk factors for identifying serious disorders causing low back pain, were documented by the GPs for each patient [32].

### Statistical analysis

We calculated summary statistics (mean, median, standard deviation, percentiles, frequencies, and percentages) for each of the two study groups, i.e., CWP and CLBP.

Testing for differences in outcome variables between CWP and CLBP patients was done using chi-square tests (for categorical data with expected cell frequencies of n ≥ 5), Fisher’s exact tests (for categorical data with expected cell frequencies of n > 5), t-tests accounting for unequal variances (Welsh tests), and Mann–Whitney U tests (for ordinal data).

## Results

### Enrollment of general practitioners and patients

Fifty-eight of the 284-invited GPs (20.4%) participated in the study. The majority of them were male (58.6%), averaged 53 years old, and worked in practices with differing sizes from both urban and rural locations and having varying organizational structures (solo/group practices).

During the recruitment period, 746 eligible patients were asked to participate. Seventy-six patients declined participation at once, and fifteen patients withdrew consent when seeing the questionnaire. Therefore, 655 participants completed the questionnaire. We excluded eight additional subjects from analyses because they did not report lower back pain on the pain drawing. A total of, 647 patients were analysed.

### Total group characteristics

A quarter of all participating CLBP patients (163, 25.2%) met CWP criteria in the pain drawing. The majority (52.1%) of all CLBP patients reported having back pain for more than ten years. On average, the participating patients were 56.5 years old. The majority was female (61.6%), married (65.1%), and lived in a two-person household (48.6%). Half of the participants are working (50.5%). Of the non-working participants, 75.2% were retired.

### Differences between CLBP and CWP patients

#### Sociodemographic data

A higher fraction of females was observed among CWP patients compared to CLBP patients (CWP 71.8% vs. CLBP 57.6%). CWP patients were less working (CWP 52.8% vs. CLBP 43.6%). Reasons for not working were comparable between groups. CWP patients more frequently reported a current pension application (CWP 14.1% vs. CLBP 5.2%) and having a degree of disability (CWP 54.2% vs. CLBP 38.5%). Other sociodemographic parameters such as age, marital status, education and reasons for not working, showed no differences between the two groups. For a detailed description of the sociodemographic data see Table 1.

**Table 1:** Sociodemographic data: differences between CLBP and CWP (n = 647)

	**CLBP**	**CWP**	**p**			
			**Chi-square test**	**Fisher’s exact test**	**Welch’s t-test**	**Mann-Whitney U test**
**Sex** [no. (%)]	n = 484	n = 163	0.002	-	-	-
Female	279 (57.6)	117 (71.8)				
**Age** [years: mean (SD)]	n = 484	n = 163	-	-	0.573	-
	56.3 (14.4)	57.0 (12.6)				
**Marital status** [no. (%)]	n = 484	n = 163	0.128	-	-	-
Single	61 (12.6)	22 (13.5)				
Married	326 (67.4)	95 (58.3)				
Divorced	54 (11.2)	28 (17.2)				
Widowed	43 (8.9)	18 (11.0)				
**Level and years of education** [no. (%)]	n = 482	n = 163	-	0.425	-	-
13/12 years	70 (14.5)	31 (19.0)				
10 years	148 (30.7)	45 (27.6)				
9 years	251 (52.1)	81 (49.7)				
Other graduation	11 (2.3)	4 (2.5)				
No qualification	2 (0.4)	2 (1.2)				
**Employment status** [no. (%)]	n = 481	n = 163	0.046	-	-	-
Working (full or part-time)	254 (52.8)	71 (43.6)				
**Reasons for not working** [no. (%)]	n = 223	n = 92	-	0.796	-	-
Keeping house	35 (15.7)	13 (14.1)				
Retired	167 (74.9)	70 (76.1)				
Unemployed	18 (8.1)	9 (9.8)				
Other	3 (1.3)	0 (0)				
**Applied for pension** [no. (%)]	n = 401	n = 128	0.002	-	-	-
Yes	21 (5.2)	18 (14.1)				
**Degree of disability** [no. (%)]	n = 457	n = 153	0.001	-	-	-
Yes	176 (38.5)	83 (54.2)				

#### Pain characteristics

The majority of CWP patients (61.3%) and 49.0% in the CLBP group reported long-lasting pain for more than ten years. While CLBP patients tended to have more pain attacks with pain-free time periods, CWP subjects suffered more frequently from permanent pain. With regards to the severity of chronic pain, we did not find significant differences between the groups. Similarly, no differences were found for the social support scale of the MPI or for the frequency of red flags between CWP and CLBP patients. More details about the pain characteristics are shown in Table 2.

**Table 2:** Pain anamnesis: differences between CLBP and CWP (n = 647)

	**CLBP**	**CWP**	**p**			
			**Chi-square test**	**Fisher’s exact test**	**Welch’s t-test**	**Mann-Whitney U-Test**
**Pain in the following areas** [no. (%)]	n = 484	n = 163				
Head	30 (6.2)	51 (31.3)	>0.001	-	-	-
Cervical spine	161 (33.3)	125 (76.7)	>0.001	-	-	-
Thoracic spine	198 (40.9)	115 (70.6)	>0.001	-	-	-
Lumbar spine	484 (100)	163 (100)	-	-	-	-
Sternum	12 (2.5)	15 (9.2)	0.001	-	-	-
Arm – left	76 (15.7)	141 (86.5)	>0.001	-	-	-
Arm – right	97 (20.0)	148 (90.8)	>0.001	-	-	-
Leg – left	160 (33.1)	140 (85.9)	>0.001	-	-	-
Leg – right	169 (34.9)	145 (89.0)	>0.001	-	-	-
Stomach	67 (13.8)	69 (42.3)	>0.001	-	-	-
**Number of pain areas** [mean (SD)]	n = 484	n = 163	-	-	-	>0.001
	3.0 (1.6)	6.8 (1.5)				
**Body mass index** [mean (SD)]	n = 472	n = 162	-	-	0.433	-
	28.1 (5.8)	28.6 (5.9)				
**First time of back pain** [no (%)]	n = 484	n = 163	-	-	-	>0.001
Since > 1 year ago	70 (14.5)	9 (5.5)				
1-2 years ago	41 (8.5)	6 (3.7)				
2-5 years ago	67 (13.8)	20 (12.3)				
5-10 years ago	69 (14.3)	28 (17.2)				
> 10 years ago	237 (49.0)	100 (61.3)				
**Back pain frequency** [no (%)]	n = 480	n = 163	-	-	-	>0.001
Few times per year	40 (8.3)	1 (0.6)				
Few times per month	47 (9.8)	10 (6.1)				
Several times per week	89 (18.5)	21 (12.9)				
One time daily	16 (3.3)	2 (1.2)				
Several times per day	99 (20.6)	27 (16.6)				
Permanent	189 (39.4)	102 (62.6)				
**Pain distribution** [no (%)]	n = 480	n = 163	0.001	-	-	-
Permanent pain with slight variations	98 (20.4)	42 (25.8)				
Permanent pain with higher variations	142 (29.6)	57 (35.0)				
Pain attacks, between pain free	164 (34.2)	28 (17.2)				
Pain attacks, between pain	76 (15.8)	36 (22.1)				
**Duration of pain attacks** [no (%)]	n = 232	n = 62	-	-	-	0.013
Seconds/minutes	41 (17.7)	6 (9.7)				
Hours	78 (33.6)	22 (35.5)				
Days	36 (15.5)	12 (19.4)				
Longer than 3 days	39 (16.8)	7 (11.3)				
Longer than 1 week	38 (16.4)	15 (24.2)				
**Red flags** [no (%)]	n = 479	n = 161	0.224	-	-	-
Positive	89 (18.6)	37 (23.0)				
**MPI - social support subscale** [mean (SD)]	n = 475	n = 141	-	-	0.234	-
	4.8 (1.9)	4.6 (1.9)				
**Graded Chronic Pain (von Korff Index)** [no (%)]	n= 447	n = 148	-	-	-	0.109
0	0 (0)	0 (0)				
1	72 (16.1)	11 (7.4)				
2	109 (24.4)	39 (26.4)				
3	131 (29.3)	50 (33.8)				
4	135 (30.2)	48 (32.4)				
**Gut feeling: Will the pain go away?** [no (%)]	n = 464	n = 156	>0.001	-	-	-
Yes	228 (49.1)	48 (30.8)				

#### Health care

CWP patients [mean (SD) 7.3 (4.1)] received a significantly higher number of different therapeutic strategies compared to CLBP patients [mean (SD) 5.7 (3.6)]. However, we found no differences in the number of GP consultations, the number of different doctors consulted, and stays in hospital during a six-month period prior to recruitment.

#### Comorbidities and psychosomatic symptoms

In general, the CWP group showed a higher number of comorbidities referring to a higher frequency of ulcer/stomach disease, kidney disease, osteoarthritis/degenerative arthritis, rheumatoid arthritis, and depression. In comparison to CLBP patients, CWP patients suffered more often from psychosomatic symptoms. All data are shown in Table 3.

**Table 3:** Comorbidities + psychosomatic symptoms: differences between CLBP and CWP (n = 647)

	**CLBP**	**CWP**	**p**			
			**Chi-square test**	**Fisher’s exact test**	**Welch’s t-test**	**Mann-Whitney U-Test**
**Self-Administered Comorbidity Questionnaire (SACQ)**						
**Disease areas (problems)** [no (%)]						
High blood pressure (n = 632)	n = 472	n = 160	0.079	-	-	-
	210 (44.5)	84 (52.5)				
Heart diseases (n = 611)	n = 460	n = 151	0.489	-	-	-
	57 (12.4)	22 (14.6)				
Asthma (n = 608)	n = 458	n = 150	0.730	-	-	-
	65 (14.2)	23 (15.3)				
Chronic obstructive pulmonary disease (n = 594)	n = 450	n = 144	0.862	-	-	-
	46 (10.2)	14 (9.7)				
Ulcer or stomach diseases (n = 605)	n = 455	n = 150	0.014	-	-	-
	77 (16.9)	39 (26.0)				
Diabetes (n = 609)	n = 462	n = 147	0.763	-	-	-
	61 (13.2)	18 (12.2)				
High blood lipid level (n = 607)	n = 454	n = 153	0.272	-	-	-
	130 (28.6)	51 (33.3)				
Kidney diseases (n = 602)	n = 454	n = 148	0.011	-	-	-
	33 (7.3)	21 (14.2)				
Osteoarthitis, degenerative arthritis (n = 619)	n = 462	n = 157	>0.001	-	-	-
	192 (41.6)	96 (61.1)				
Rheumatoid arthritis (n = 591)	n = 445	n = 146	0.005	-	-	-
	48 (10.8)	29 (19.9)				
Osteoporosis (n = 597)	n = 447	n = 150	0.454	-	-	-
	44 (9.8)	18 (12.0)				
Cancer disease (n = 600)	n = 453	n = 147	0.287	-	-	-
	33 (7.3)	7 (4.8)				
Depression (n = 598)	n = 456	n = 142	>0.001	-	-	-
	84 (18.4)	47 (33.1)				
Other psychiatric diseases (n = 596)	n = 449	n = 147	0.354	-	-	-
	54 (12.0)	22 (15.0)				
Number of diagnosed problems [mean (SD)]	n = 383	n = 118				
	2.2 (1.9)	2.9 (2.1)	-	-	-	0.001
Number of treated problems [mean (SD)]	n = 366	n = 107				
	1.3 (1.5)	1.8 (1.9)	-	-	-	0.026
Summary score [mean (SD)]	n = 331	n = 102				
	3.5 (3.6)	5.0 (4.6)	-	-	0.003	-
Symptom Checklist-90-R	n = 430	n = 143				
Summation of all items [mean (SD)]	10.0 (5.9)	15.0 (7.6)	-	-	>0.001	-
Mean of all items [mean (SD)]	0.8 (0.5)	1.3 (0.6)	-	-	>0.001	-
Number of symptoms [mean (SD)]	5.6 (2.8)	7.8 (3.0)	-	-	>0.001	-

## Discussion

To our knowledge, this project is the largest study to date which analyzes differences between chronic localized low back pain and chronic widespread pain in primary care settings.

A quarter of all included CLBP patients satisfy the ACR criteria for CWP. Altogether, CWP patients are predominantly female and showed more psychosomatic symptoms and comorbidities than CLBP patients.

The observed high prevalence of CWP (25.2%) is in accordance with the findings of Nordeman et al., who reported a CWP prevalence of 28% in females in primary care settings [9]. The slightly higher prevalence in the study of Nordeman et al. can be explained by the female sample, which is associated with higher risk for CWP [7,11].

Apart from the apparently higher proportion of females in the CWP group, which confirms similar findings of other investigators [7,11], we found no group differences in other sociodemographic data. Again, this observation is in accordance with the findings of Nordeman et al. They found no significant differences in the following sociodemographic parameters: age, nationality, education, and social status between female CLBP and CWP patients.

In contrast, a population-based study from Bergman et al. found a lower educational and social status among CWP patients [11]. There is no clear reason for this contrast. A possible explanation could be the different setting of the populations in the study (primary care vs. population based) and that Bergman et al. compared CWP versus chronic regional pain in general.

The role of somatization processes in CWP is still unclear. In the literature, two hypotheses are discussed: First, somatization could be a consequence of CWP. Second, CWP could be a manifestation of the process of somatization [11]. We only assessed typical physical symptoms which accompany functional dysfunctions; these symptoms could also arise in somatic disorders. The increased prevalence of these symptoms in the CWP group emphasizes a slight association between CWP and the process of somatization [33]. Although we cannot make any causal attributions due to the cross-sectional nature of our data, population-based cohort studies showed somatic symptoms to be a predictive factor for the onset of CWP [34,35]. This supports the hypothesis: CWP is a manifestation of the somatization process.

In comparison to individuals without back pain [8], our total study population has an increased prevalence of comorbidities. The increased rates of osteoarthritis and degenerative arthritis, rheumatoid arthritis, depression, ulcer or stomach diseases, and kidney diseases among CWP patients correspond with the observations of Kato et al. [15]. They extracted CWP cases and their healthy siblings from the Swedish Twin Registry and assessed their comorbidities. A comparative analysis showed that CWP patients have an increased odds ratio for having joint pain, depressive symptoms, irritable bowel syndrome, and chronic fatigue syndrome.

The increased rates of ulcer/stomach and kidney diseases in our CWP sample might be related to a higher consumption of non-steroidal anti-inflammatory drugs (NSAID). Furthermore, ulcer and stomach diseases are typical symptoms of somatization processes as discussed above [36]. Apart from this, depression is a well-known comorbidity of CWP [36]. GPs should immediately consider the parallel comorbidities while treating CWP patients.

The definition of chronic widespread pain comprises duration and local extension of the pain [1]. It is an operational definition on a symptomatic level which does not allow for any conclusions with respect to the aetiology of CWP. Consequently, it is impossible to differentiate between patients with multiple regional pain and patients who experience a generalization of their pain by somatic causes (e.g. arthrosis in joint, hallux valgus). With a sensitivity of 98% and a specificity of 31% of the current definition from the ACR [37], it is most likely that there is a high proportion of patients in our sample that suffers from multiple regional pain since the majority of patients in general practices are older and suffer from multimorbidity, including degenerative diseases. However, based on epidemiological findings, Macfarlane postulated that a differentiation of these would be artificial and obscuring important aspects of aetiology [37].

In discussing group differences, we must emphasize that our study population is comprised of patients with a severe disease pattern (52%: pain > 10 years). Therefore, we assume that our subgroups do not greatly differ due to the already extreme nature of our subjects. This might also be a reason for the similarity of CLBP and CWP patients regarding pain severity according to von Korff [28]. Both groups showed high rates of “pain related disability”, which forms the upper two grades of severity independent of the dimension “pain intensity”.

With respect to methodological limitations, we have to think of selection bias in our study. In our study, some participations denied participation due to the long, demanding questionnaire. Selective recruitment might have occurred because general practitioners might forget to recruit patients due to a high workload. Furthermore, it might be possible that GPs are more likely to recruit special cases (e.g., patients with higher disease severity or unique personality). This might reduce the external validity of our results.

Although a GP recruitment rate of about 20% is normal in our research field, we should consider selection bias. We do not know if non-participating GPs have CLBP patients with characteristics different than the participating GPs. However, regarding sociodemographic data and practice characteristics, the GP sample seems to be representative for the region of northern Hessen.

## Conclusion

Our primary finding is the high CWP prevalence among CLBP patients in primary care settings. Therefore, CWP is a frequent and particularly severe pain syndrome. Although it is impossible to identify CWP patients with the help of typical sociodemographic profiles, we showed that CWP patients are more likely to suffer from psychosomatic symptoms and comorbidities than CLBP patients. These comorbidities must be considered when a GP makes treatment decisions based on the fact that a patient has CWP. Further research is needed to clarify the role of somatization in conjunction with chronic widespread pain to infer the direction of causality.

## Abbreviations

ACR: American College of Rheumatology; CLBP: Chronic low back pain; CLP: Chronic localized pain; CWP: Chronic widespread pain; GP: General practitioner; MPI: West Haven-Yale Multidimensional Pain Inventory; SCL-90-R: Symptom Checklist-90-R.

## Competing interests

Annika Viniol, Nikita Jegan, Markus Brugger, Konstantin Strauch, Jürgen Barth, Erika Baum and Corinna Leonhardt do not state any financial or non-financial conflicts of interests.

Annette Becker was a consultant for Grünenthal GmbH, from whom she has also received a speaker’s fee.

## Authors’ contributions

AV collected and analyzed data and wrote the manuscript. NJ collected and analyzed data. MB and KS gave statistical support and planned the study. JB and EB planned and revised the study design. CL and AB planned the study, discussed the results, and revised the manuscript critically. All authors edited the drafted version of the manuscript. All authors read and approved the final manuscript.

## Pre-publication history

The pre-publication history for this paper can be accessed here:

http://www.biomedcentral.com/1471-2474/14/351/prepub

## References

[B1] Wolfe F Smythe HA Yunus MB Bennett RM Bombardier C Goldenberg DL Tugwell P Campbell SM Abeles M Clark P The American College of Rheumatology 1990 Criteria for the Classification of Fibromyalgia. Report of the Multicenter Criteria Committee Arthritis Rheum 1990 14 160 172 10.1002/art.1780330203 2306288

[B2] Natvig B Bruusgaard D Eriksen W Localized low back pain and low back pain as part of widespread musculoskeletal pain. Two different disorders? A cross-sectional population study J Rehabil Med 2001 14 21 25 10.1080/165019701300006498 11480465

[B3] Gerdle B Bjork J Coster L Henriksson K Henriksson C Bengtsson A Prevalence of widespread pain and associations with work status. A population study BMC Musculoskelet Disord 2008 14 102 10.1186/1471-2474-9-102 18627605 PMC2488345

[B4] Hunt IM Silman AJ Benjamin S McBeth J Macfarlane GJ The prevalence and associated features of chronic widespread pain in the community using the ‘Manchester’ definition of chronic widespread pain Rheumatology (Oxford) 1999 14 275 279 10.1093/rheumatology/38.3.275 10325667

[B5] Macfarlane GJ Pye SR Finn JD Wu FC Silman AJ Bartfai G Boonen S Casanueva F Forti G Giwercman A Han TS Huhtaniemi IT Kula K Lean ME O’Neill TW Pendleton N Punab M Vanderschueren D Investigating the determinants of international differences in the prevalence of chronic widespread pain. Evidence from the European Male Ageing Study Ann Rheum Dis 2009 14 690 695 10.1136/ard.2008.089417 18653627

[B6] Croft P Rigby AS Boswell R Schollum J Silman A The prevalence of chronic widespread pain in the general population J Rheumatol 1993 14 710 713 8496870

[B7] Cho NH Kim I Lim SH Kim HA Prevalence of widespread pain and its influence on quality of life. Population study in Korea J Korean Med Sci 2012 14 16 21 10.3346/jkms.2012.27.1.16 22219608 PMC3247768

[B8] Hüppe A Raspe H Amplifizierte Rückenschmerzen und Komorbidität in der Bevölkerung Schmerz 2009 14 275 283 19308463 10.1007/s00482-009-0783-8

[B9] Nordeman L Gunnarsson R Mannerkorpi K Prevalence and characteristics of widespread pain in female primary health care patients with chronic low back pain Clin J Pain 2012 14 65 72 10.1097/AJP.0b013e318223622c 21677567

[B10] Spaeth M Epidemiology, costs, and the economic burden of fibromyalgia Arthritis Res Ther 2009 14 117 10.1186/ar2715 19591654 PMC2714132

[B11] Bergman S Psychosocial aspects of chronic widespread pain and fibromyalgia Disabil Rehabil 2005 14 675 683 10.1080/09638280400009030 16012060

[B12] McBeth J Nicholl BI Cordingley L Davies KA Macfarlane GJ Chronic widespread pain predicts physical inactivity. Results from the prospective EPIFUND study Eur J Pain 2010 14 972 979 10.1016/j.ejpain.2010.03.005 20400346 PMC3161181

[B13] Overland S Harvey SB Knudsen AK Mykletun A Hotopf M Widespread pain and medically certified disability pension in the Hordaland Health Study Eur J Pain 2012 14 611 620 10.1016/j.ejpain.2011.08.005 22396089

[B14] Kadam UT Thomas E Croft PR Is chronic widespread pain a predictor of all-cause morbidity? A 3 year prospective population based study in family practice J Rheumatol 2005 14 1341 1348 15996075

[B15] Kato K Sullivan PF Evengard B Pedersen NL Chronic widespread pain and its comorbidities. A population-based study Arch Intern Med 2006 14 1649 1654 10.1001/archinte.166.15.1649 16908799

[B16] Palm O Moum B Jahnsen J Gran JT Fibromyalgia and chronic widespread pain in patients with inflammatory bowel disease: a cross sectional population survey J Rheumatol 2001 14 590 594 11296964

[B17] Benjamin S Morris S McBeth J Macfarlane GJ Silman AJ The association between chronic widespread pain and mental disorder. A population-based study Arthritis Rheum 2000 14 561 567 10.1002/1529-0131(200003)43:3<561::AID-ANR12>3.0.CO;2-O 10728749

[B18] Andersson HI Increased mortality among individuals with chronic widespread pain relates to lifestyle factors. A prospective population-based study Disabil Rehabil 2009 14 1980 1987 10.3109/09638280902874154 19874076

[B19] Andersson HI Ejlertsson G Leden I Rosenberg C Characteristics of subjects with chronic pain, in relation to local and widespread pain report. A prospective study of symptoms, clinical findings and blood tests in subgroups of a geographically defined population Scand J Rheumatol 1996 14 146 154 10.3109/03009749609080005 8668957

[B20] Raphael KG Marbach JJ Klausner J Myofascial face pain. Clinical characteristics of those with regional vs. widespread pain J Am Dent Assoc 2000 14 161 171 10680383 10.14219/jada.archive.2000.0143

[B21] Willweber-Strumpf A Zenz M Bartz D Epidemiologie chronischer Schmerzen. Eine Befragung in 5 Facharztpraxen in Bochum Schmerz 2000 14 84 91 10.1007/s004820050226 12800044

[B22] Kühlein T Laux G Gutscher A Szecsenyi J Kontinuierliche Morbiditätsregistrierung in der Hausarztpraxis: Vom Beratungsanlass zum Beratungsergebnis 2008 München: Urban & Vogel GmbH

[B23] Viniol A Jegan N Leonhardt C Strauch K Brugger M Barth J Baum E Becker A Study protocol: transition from localized low back pain to chronic widespread pain in general practice: identification of risk factors, preventive factors and key elements for treatment - a cohort study BMC Musculoskelet Disord 2012 14 77 10.1186/1471-2474-13-77 22630134 PMC3409020

[B24] Pfau DB Rolke R Nickel R Treede R Daublaender M Somatosensory profiles in subgroups of patients with myogenic temporomandibular disorders and fibromyalgia syndrome Pain 2009 14 72 83 10.1016/j.pain.2009.08.010 19767146

[B25] Deutsche Gesellschaft zum Studium des Schmerzes (DGSS) Deutscher Schmerzfragebogen Köln

[B26] Kerns R Turk D Rudy T The West Haven-Yale Multidimensional Pain Inventory (WHYMPI) Pain 1985 14 345 356 10.1016/0304-3959(85)90004-1 4088697

[B27] Flor H Rudy TE Birbaumer N Streit B Schugens MM Zur Anwendbarkeit des West Haven-Yale Multidimensional Pain Inventory im deutschen Sprachraum. Daten zur Reliabilität und Validität des MPI-D Schmerz 1990 14 82 87 10.1007/BF02527839 18415223

[B28] von Korff M Dworkin S Resche L Graded chronic pain status an epidemiologic evaluation Pain 1990 14 179 291 10.1016/0304-3959(90)91125-3 2326094

[B29] Klasen BW Hallner D Schaub C Willburger R Hasenbring M Validation and reliability of the German version of the Chronic Pain Grade questionnaire in primary care back pain patients Psychosoc Med 2004 14 Doc07 19742049 PMC2736479

[B30] Sangha O Stucki G Liang MH Fossel AH Katz JN The Self-Administered Comorbidity Questionnaire. A new method to assess comorbidity for clinical and health services research Arthritis Rheum 2003 14 156 163 10.1002/art.10993 12687505

[B31] Franke G Stäcker K Reliabilität und Validität der Symptom-Check-Liste (SCL-90-R; Derogatis 1986) bei Standardreihenfolge versus inhaltshomogener Itemblockbildung Diagnostica 1995 14 349 373

[B32] Tulder M Becker A Bekkering T Breen A Gil del Real MT Hutchinson A Koes B Laerum E Malmivaara A Chapter 3 European guidelines for the management of acute nonspecific low back pain in primary care Eur Spine J 2006 14 s169 10.1007/s00586-006-1071-2 16550447 PMC3454540

[B33] Lipowski ZJ Somatization: the concept and its clinical application Am J Psychiatry 1988 14 1358 1368 3056044 10.1176/ajp.145.11.1358

[B34] McBeth J Macfarlane GJ Hunt IM Silman AJ Risk factors for persistent chronic widespread pain. A community-based study Rheumatology (Oxford) 2001 14 95 101 11157148 10.1093/rheumatology/40.1.95

[B35] Gupta A Silman AJ Ray D Morriss R Dickens C Macfarlane GJ Chiu YH Nicholl B McBeth J The role of psychosocial factors in predicting the onset of chronic widespread pain. Results from a prospective population-based study Rheumatology (Oxford) 2007 14 666 671 17085772 10.1093/rheumatology/kel363

[B36] Kroenke K Mulitisomatoform disorder. An alternative to undifferentiated somatoform disorder for the somatizing patient in primary care Arch Gen Psychiatry 1997 14 352 358 10.1001/archpsyc.1997.01830160080011 9107152

[B37] Macfarlane GJ Generalized pain, fibromyalgia and regional pain: an epidemiological view Baillieres Best Pract Res Clin Rheumatol 1999 14 403 414 10.1053/berh.1999.0029 10562370

